# Supplementary material for: Symmetry mismatch-driven perpendicular magnetic anisotropy for perovskite/brownmillerite heterostructures
Source: Nat Commun. 2018 May 15;9:1923. doi: 10.1038/s41467-018-04304-7 (PMC5953968; doi:10.1038/s41467-018-04304-7)
Supplement: Supplementary file 1 — Supplementary Information [file 41467_2018_4304_MOESM1_ESM.pdf]

Supplementary Information for

Symmetry mismatch-driven perpendicular magnetic anisotropy for perovskite/brownmillerite heterostructures

By Zhang et al.

LCO(6nm)/LSMO(6nm)/LCO(6nm) trilayers

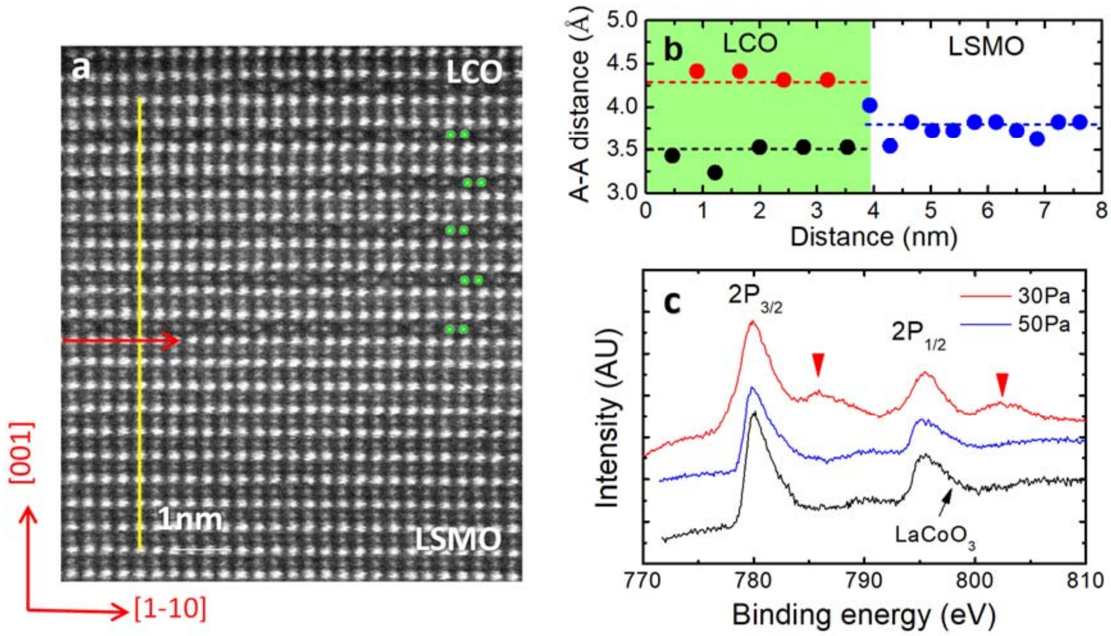

Supplementary Figure 1. A-site atomic separation around the LSMO/LCO interface. (a) A high-angle annular dark-field (HAADF) image of the cross sections of the LCO(5nm)/LSMO(5nm)/LCO(5nm) heterostructure, recorded along the [110] zone. Arrow marks the LSCO/LCO interface. (b) A-A (A=La or Sr) distance obtained along the yellow line in (a). Dashed lines indicate the results of density functional theory calculation for the brownmillerite LCO film and the perovskite LSMO film grown on STO substrates. (c) Spectra of X-ray photoelectron spectroscopy (XPS) of the Co ions in the LCO(6nm)/LSMO(6nm)/LCO(6nm) trilayers prepared under the oxygen pressures of 30 Pa and 50 Pa, respectively. Obvious shake up satellite peaks (marked by two red triangles) are observed for the former sample but not for the latter sample. This result confirms the presence of considerable Co^{2+} in the former trilayers, for which LCO is expected to be brownmillerite-structured. For comparison, the XPS spectrum of perovskite $LaCoO_3$ film (6 nm in thickness) is also presented.

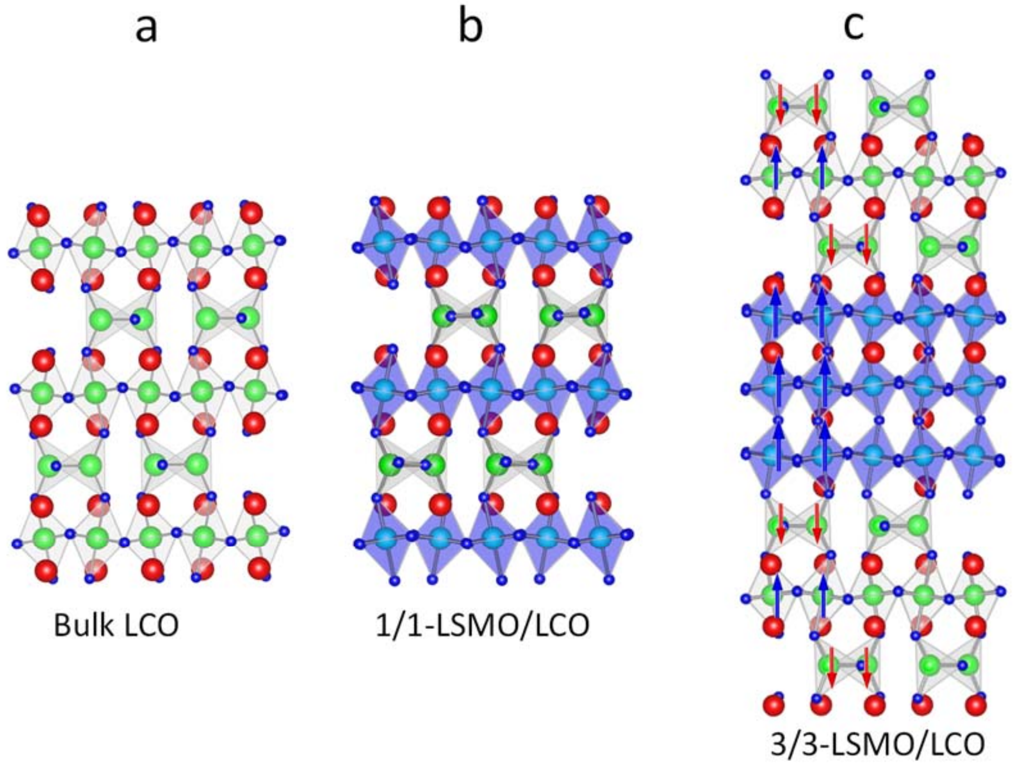

Supplementary Figure 2. Atomic structures of density functional theory calculations. (a) Bulk brownmillerite LCO. (b) 1/1-LSMO/LCO. (c) 3/3-LSMO/LCO. We studied bulk brownmillerite $LaCoO_{2.5}$ (Figure 2a), N/N-perovskite/brownmillerite interfaces of LSMO/$LaCoO_{2.5}$ with N=1 and 3 (Figures 2b and 2c). Bulk $LaCoO_{2.5}$ is constructed from the brownmillerite $SrCoO_{2.5}$ by replacing Sr with La. The brownmillerite structure can be viewed as perovskite structure with ordered oxygen vacancies, which leads to alternately stacking of CoO_4 tetrahedra and CoO_6 octahedra. Density functional theory (DFT) calculated lattice constant without any constrain is a= 5.349 Å, b= 5.713 Å, c= 15.542 Å. The lattice anisotropy between a and b arises from the CoO_4 tetrahedral chain along a orientation. To simulate the strain effect from the $SrTiO_3$ substrate, we fixed a and b to 3.905 Å, the lattice constant of $SrTiO_3$. We found that the La-La distance around the CoO_4 is 4.180 Å, which corresponds to the dark stripe in the STEM, while the La-La distance around the CoO_6 is 3.364 Å, which corresponds to the bright stripe in the STEM. Moreover, La atoms are not perfectly aligned in the out-of-plane orientation but form zigzag patterns, in agreement with experimental observation. Along (110) orientation, we could see Co-Co pair with a shorter distance of 2.148 Å in contrast to the long distance of 3.364 Å between two pairs.

To highlight the effect of perovskite and brownmillerite interface, we constructed the 1/1-P/B interface of LCO/LSMO by replacing the Co atom in the CoO_6 octahedral by a Mn

atom (Figure 2b). The structure can be viewed as alternately stacking of CoO_4 tetrahedral and MnO_6 octahedral. We substitute part of La atoms by Sr atoms to simulate LSMO; we studied several Sr substitution and found that the main conclusion did not change. To have a better description of the experimental LSMO/LCO SLs, we increased the thickness of each layer and constructed a 3/3-P/B interface as shown in Figure 2c. It contains two P/B interfaces of the MnO_6/CoO_4 type. The optimized structures preserve the features of the brownmillerite LCO such as the La-La structural pattern and distantly separated Co-Co pair. Furthermore, at the P/B interface, there is an oxygen atom that links the CoO_4 tetrahedron and MnO_6 octahedron. Its crystal environment is very different from that of LSMO. This oxygen atom will relax towards LCO layer, leading to a ~0.5 Å La-O displacement along z direction, which is qualitatively consistent with experimental observed value of 0.6 Å. Moreover, the interfacial MnO_6 is substantially distorted. The MnO bond pointing to the interface is 0.2 Å longer than other MnO bonds. Those kinds of interfacial symmetry broken will significantly modify the electronic properties, playing the key role in magnetocrystalline anisotropy.

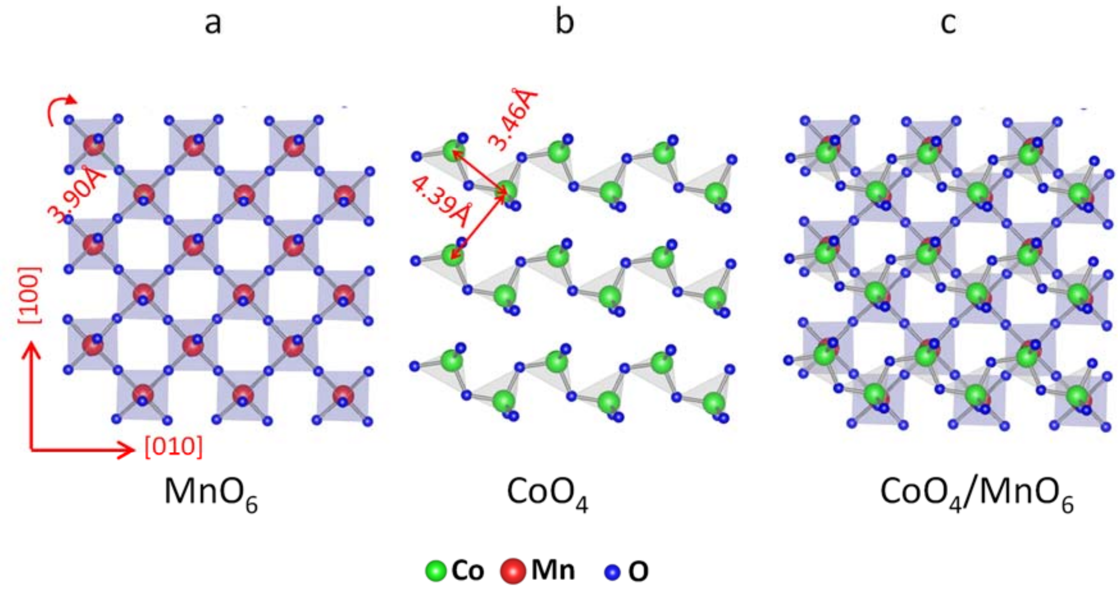

Supplementary Figure 3. Calculated lattice structures for the LSMO/LCO superlattices. (a) A single MnO_6 layer. (b) A CoO_4 single layer. (c) A CoO_4/MnO_6 stack. The Mn/Co misalignment can be clearly seen from (c). The nearest Co-Co distance is shorter in the CoO_4 chain and longer between two neighboring chains. This causes a regular misalignment of top Co and bottom Mn atoms. For example, the first row of Co atoms in (c) shows a bottom left shift while the second row displays an up left shift. Consequently, the MnO_6 octahedron has to tilt around the [1-10] axis (marked by a red arrow) and elongates along the [001] axis. Here the La/Sr atom has been dropped for clarity

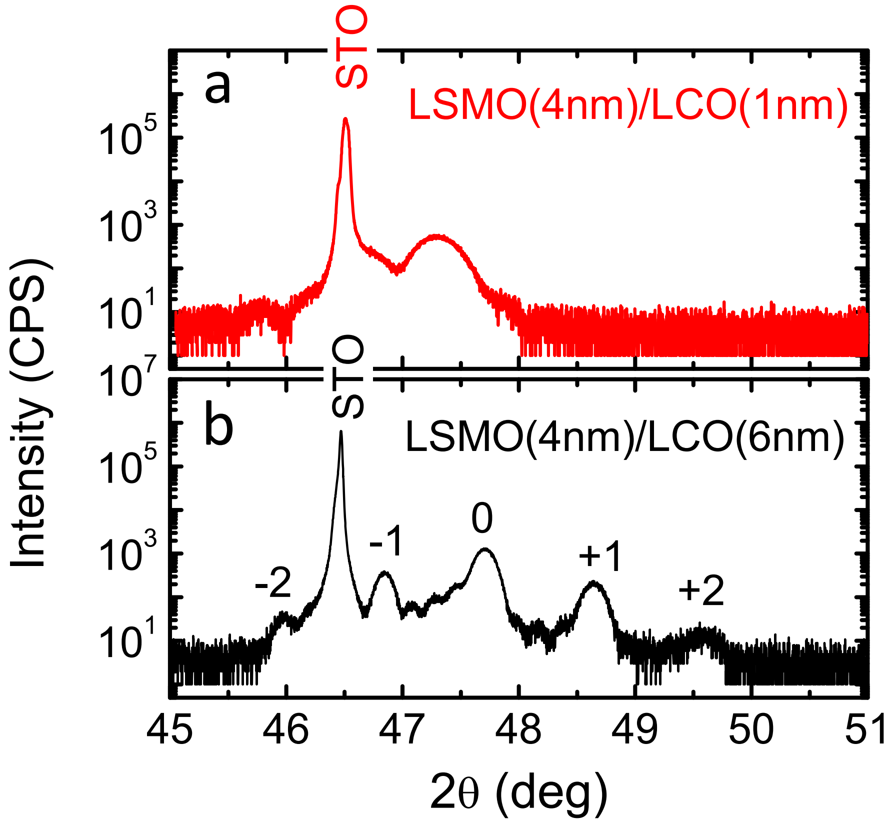

Supplementary Figure 4. X-ray diffraction patterns of the LSMO/LCO superlattices a repetition of 5. (a) LSMO(4nm)/LCO(1nm). (b) LSMO(4nm)/LCO(6nm). Satellite peaks corresponding to superstructure (labeled by numbers) and interferences due to finite film thickness can be clearly seen.

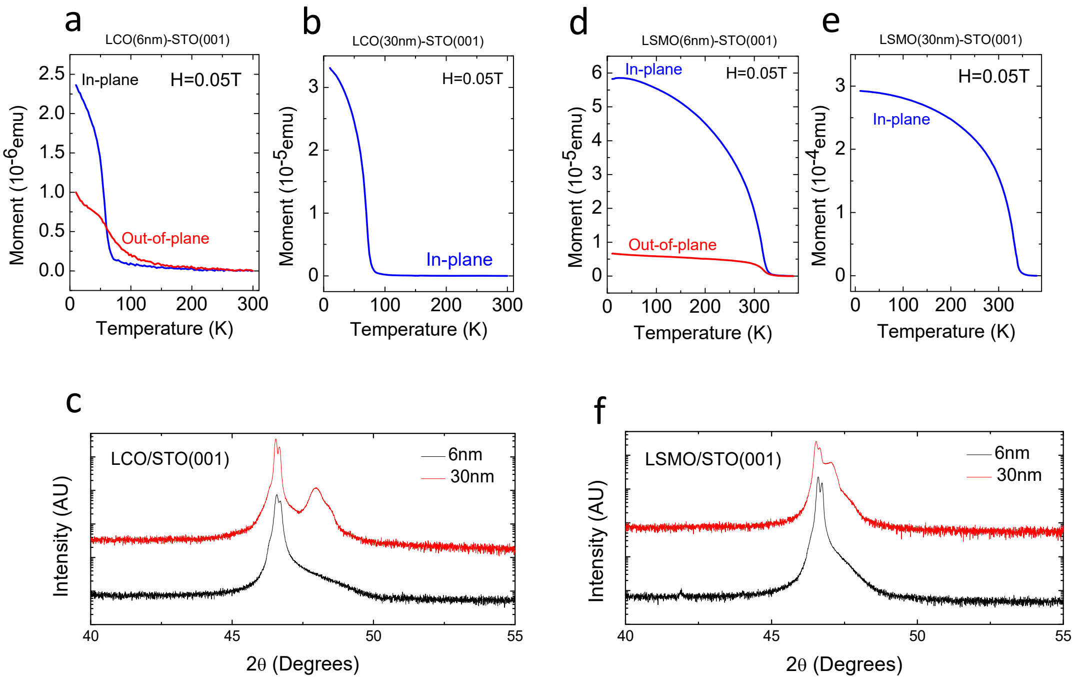

Supplementary Figure 5. Structural and magnetic properties of bared LSMO and LCO films. (a, b) Temperature dependences of the magnetic moments of bared LCO films with the thicknesses of 6 nm and 30 nm, respectively. (c) X-ray diffraction spectra of the corresponding films. (d, e) Temperature dependences of the magnetic moments of bared LSMO films with the thicknesses of 6 nm and 30 nm, respectively. (f) X-ray diffraction spectra of the corresponding films.

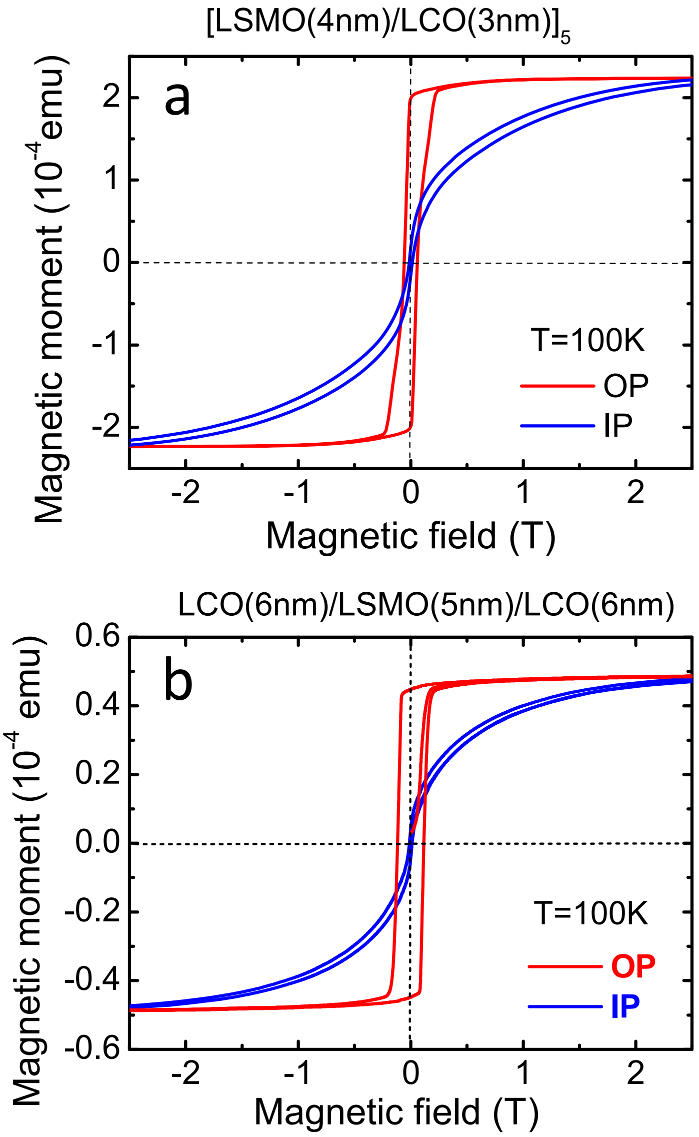

Supplementary Figure 6. Magnetic loops measured with respectively in-plane (IP) and out-of-plane (OP) applied fields. (a) LSMO(4nm)/LCO(3nm) superlattices. (b) LCO(6nm)/LSMO(5nm)/LCO(6nm) trilayers. T=100 K. Out-of-plane magnetic anisotropy is clearly seen.

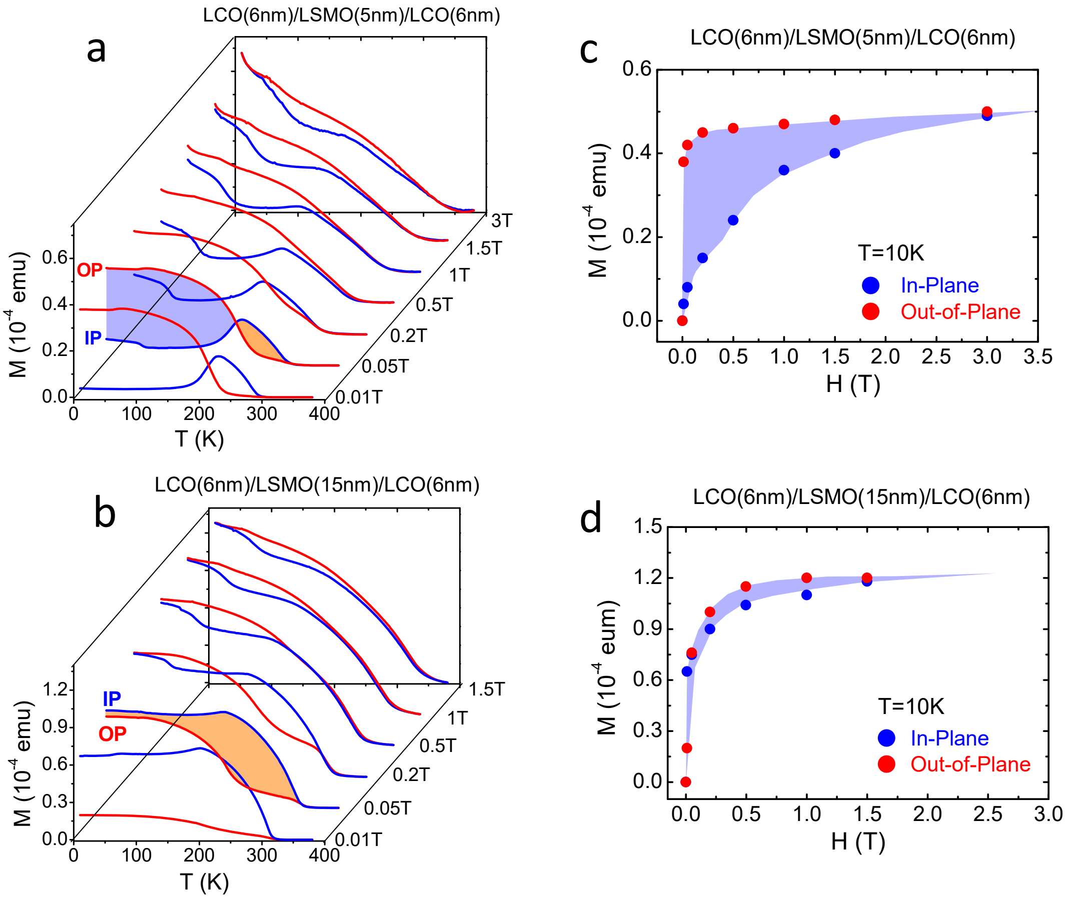

Supplementary Figure 7. Magnetic properties of the LCO)/LSMO/LCO trilayers. (a-b) Thermomagnetic curves of the LCO(6nm)/LSMO(5nm)/LCO(6nm) and LCO(6nm)/LSMO(15nm)/LCO(6nm) trilayers, collected in field-cooling mode with in-plane (IP) and out-of-plane (OP) applied fields. Purple and orange areas highlight the difference of the magnetic moments along two measuring directions. (c-d) Magnetic field dependences of the magnetic moments of the corresponding samples. T=10 K.

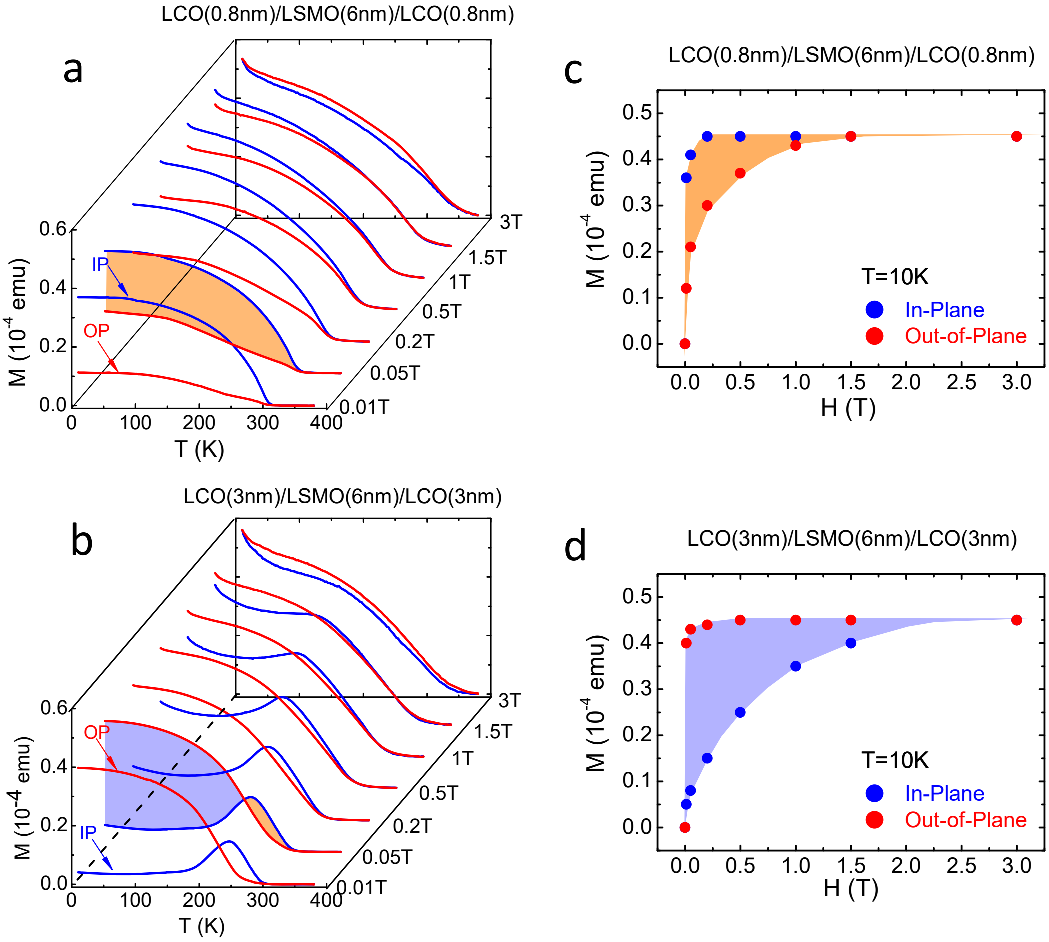

Supplementary Figure 8. Magnetic properties of the LCO)/LSMO/LCO trilayers. (a-b) Thermomagnetic curves of the LCO(0.8nm)/LSMO(6nm)/LCO(0.8nm) and LCO(3nm)/LSMO(6nm)/LCO(3nm) trilayers, collected in field-cooling mode with in-plane (IP) and out-of-plane (OP) applied fields. Purple and orange areas highlight the difference of the magnetic moments along two measuring directions. (c-d) Magnetic field dependences of the magnetic moments of these two samples. T=10 K.

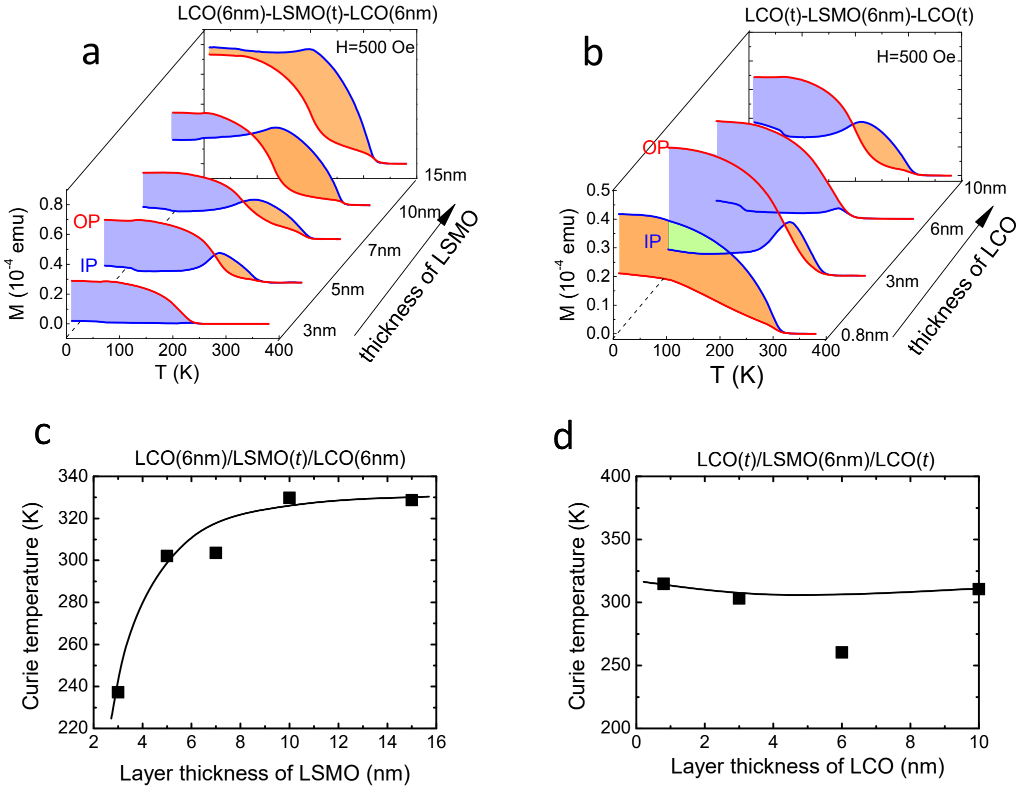

Supplementary Figure 9. Curie temperature as function of layer thickness. (a-b) Thermomagnetic curves of the LCO/LSMO/LCO trilayers with different LSMO (a) or LCO (b) layer thicknesses. IP and OP denote the in-plane and out-of-plane fields, respectively. (c-d) Curie temperatures of the corresponding samples. The anomalously low Curie temperature around t_{LCO}=6 nm is due to the fluctuation of sample preparation condition.

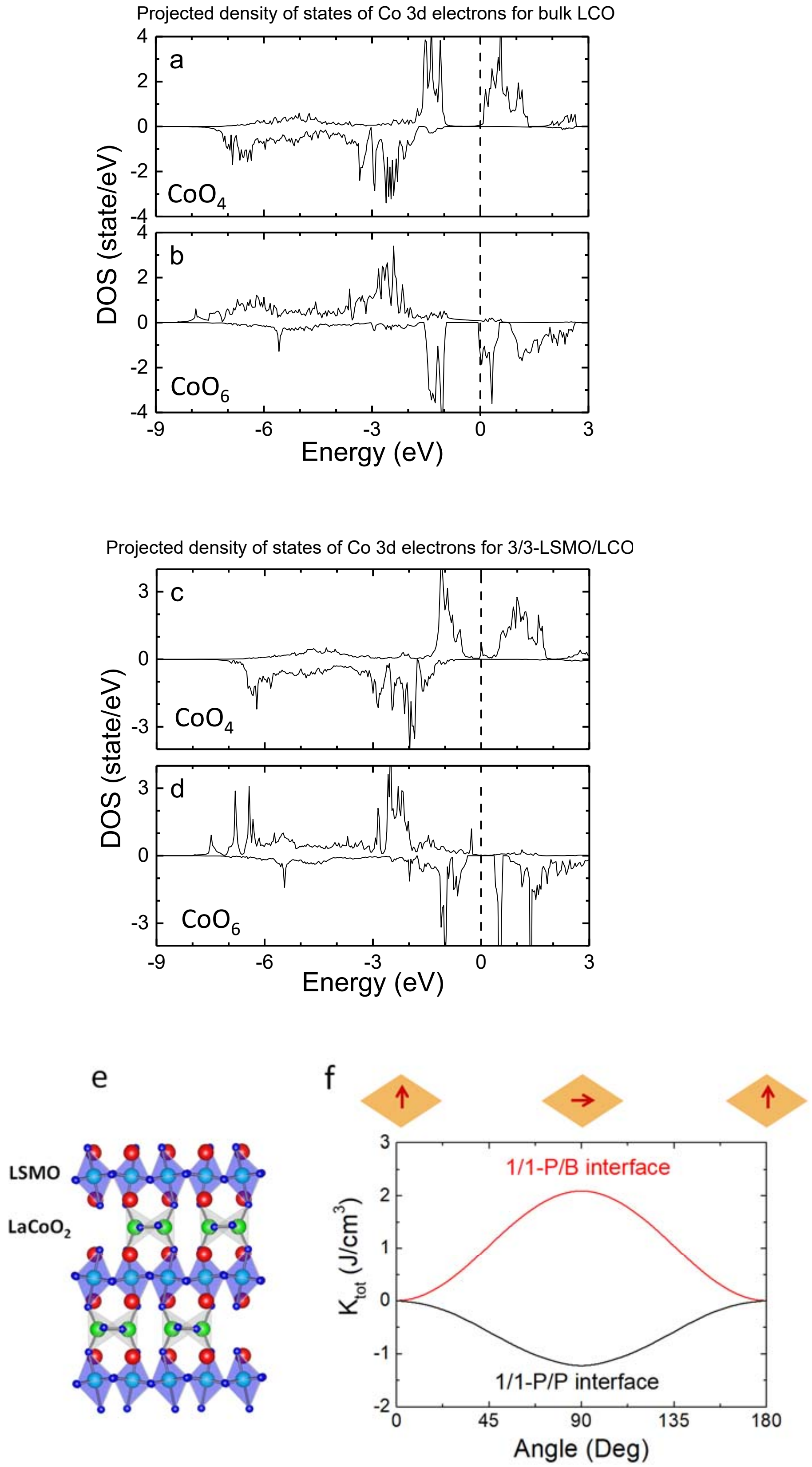

Supplementary Figure 10. Results of density functional theory calculations. (a, b) Projected density of states of Co 3d electrons for bulk LCO. (c, d) Projected density of states of Co 3d electrons for 3/3-LSMO/LCO superlattices. (e, f) Non-self-consistent DFT

calculated magnetic anisotropy with tuning magnetization angle from out-of-plane to in-plane continuously for both 1/1-P/B and 1-1/-P/P interfaces. Details of the density functional theory (DFT) calculations are as follows:

DFT calculated magnetic ground state is A-AFM for bulk brownmillerite $LaCoO_{2.5}$, i.e., spins of CoO_6 or CoO_4 are parallel, while they are antiparallel between CoO_6 and CoO_4. The local magnetic moments of the Co atom of CoO_6 and CoO_4 are 2.478 μ_B and -2.429 μ_B, respectively. Given the formal valence La^{3+} and O^{2-}, the Co valence in $LaCoO_{2.5}$ is 2+ with d^7 configuration. For a high spin configuration, it is expected to have a local magnetic moment of 3 μ_B, with five electrons occupying the majority channel and two electrons occupying minority channel. As shown in the projected DOS in Figure 10a, one spin channel in Co of CoO_4 is fully occupied. Since in a tetrahedral crystal field two e_g orbitals are lower in energy than three t_{2g} orbitals, two electrons occupy the two e_g orbitals in minority spin channel, and an energy gap is open. It leads to 3 μ_B local magnetic moment, in agreement with DFT calculated 2.478 μ_B (the discrepancy arises from hybridization with oxygen atoms). In an octahedral crystal field of CoO_6 three t_{2g} orbitals are lower in energy than that of two e_g orbitals. Thus, two electrons in minority spin channel partially occupy three t_{2g} orbitals and exhibits metallic behavior (Figure 10b). DFT calculations indicate that the valence of the two Co atoms should be 2+. Please also note that the oxygen states in CoO_4 and CoO_6 are quite different, and thus Co-O hybridization is different, which might explain the valence difference in the EELS measurement.

In comparison with the DOS of bulk $LaCoO_{2.5}$ in Figures 10a and 10b and the 3/3-P/B interface in Figures 10c and 10d, we do not see significant change of Co states as well as charge transfer across the interface. Co valence does not change too much, in agreement with experimental observation. DFT calculated magnetic ground state is FM ordering of Mn spins and A-AFM LCO of Co, while the magnetic coupling between Mn and Co is antiferromagnetic, as shown in Supplementary Figure 2c. As mentioned, e_g electrons in CoO_4 tetrahedra are more energetically favorable, while t_{2g} electrons in MnO_6 octahedra are more energetically favorable. This interfacial orbital rearrangement, together with structural distortion and symmetry broken will induce complex and unique physics in the P/B interface. For instance, d_{3z2-r2} occupation is much enhanced in the interfacial LSMO layer. Such an orbital polarization together with spin orbit coupling effect will induce out of plane magnetocrystalline anisotropy. Our DFT calculated MAE of 1/1-P/B interface is 0.78 meV/Mn at each interface. We also performed non-self-consistent DFT calculations with tuning magnetization angle from out-of-plane to in-plane continuously for both 1/1-P/B and 1-1/-P/P interfaces, as shown in Figures 10e and 10f. It confirms that the P/B interface play the key role for the perpendicular magnetism anisotropy. Increasing the thickness N to 3, the MAE reduced to 0.15 meV, which is qualitatively consistent with experimental observation.
